# Single stage repair of a complex pathology: end stage ischaemic cardiomyopathy, ascending aortic aneurysm and thoracic coarctation

**DOI:** 10.1186/1749-8090-6-152

**Published:** 2011-11-20

**Authors:** Haralabos Parissis, Bassel Al-Alao, Allan Soo, John Dark

**Affiliations:** 1 Cardiothoracic Department, Royal Victoria Hospital, BT 12 6BA, Belfast, UK; 2 Cardiopulmonary Transplantation, Freeman Hospital, Newcastle, UK

## Abstract

The not uncommon combination of ascending aortic pathology with late presenting coarctation is a difficult surgical challenge. The two stage approach is usually adopted. The necessity for cardiac transplantation adds to the complexity: a trans-sternal approach and single stage repair become mandatory.

## Introduction

A direct approach to repair thoracic coarctation may entail difficulties in the adult population.

Concurrent aortic and cardiac pathology represents a rare entity. This report describes a technique of addressing a complex pathology: a patient with an aortic coarctation, an ascending aortic aneurysm and end stage ischaemic cardiomyopathy.

**Figure 1:**
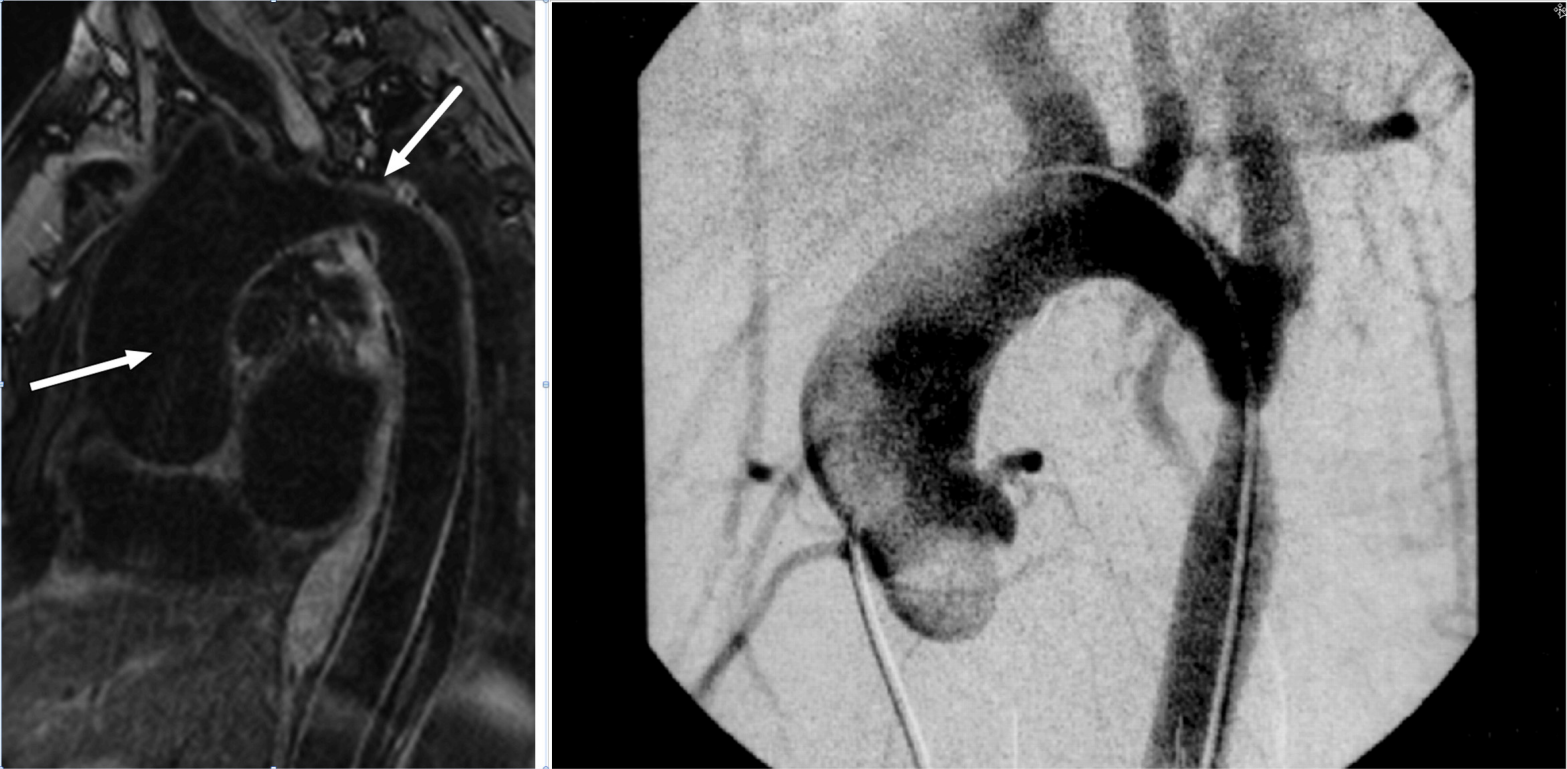
**MRI of the chest, depicting the ascending thoracic aneurysm leading to a tight coarctation of the descending aorta**. The Aortogram also delineates similar pathology.

A 58 years old male presented with tachycardia and biventricular failure. Investigations revealed a 9 cm ascending aortic aneurysm containing a dissection flap above a regurgitant bicuspid aortic valve. Despite anti-failure treatment the ejection fraction remained in the range of 15-20%. A CT scan and an MRI (Figure 1) further delineated the ascending aortic aneurysm and revealed an unsuspected but very tight coarctation with good collaterals. A subsequent attempt at coronary angiography failed because of inability to engage the grossly displaced coronary ostia. Very extensive coronary calcification, reaching out to the terminal branches of the major epicardial vessels was however revealed. The conventional procedure would have entailed aortic valve and ascending aortic replacement together with blind CABG and repair of the coarctation. Further episodes of heart failure persuaded us that transplantation was a more acceptable argument. The potential difficulties of grafting only small distal vessels in a patient who already had severe left ventricular dysfunction was a major factor in this decision. The patient was accepted on to the transplant waiting list.

Because of his ambulatory nature he remained on the waiting list whilst gradually deteriorating. He was admitted as an emergency with gross congestive heart failure some 11 months after listing. At that time repeat right heart catheter revealed a mean pulmonary artery pressure of 42 with a pulmonary artery papillary wedge of 36 and a cardiac index of less than 2. His creatinine had risen to 137 mm/l. He was admitted to hospital and treated with Dobutamine and Frusemide infusion and listed for urgent transplantation.

After a further 10 days a donor heart of a 17 year old male of acceptable weight and height became available to us. The most striking finding at the operation of the recipient was the huge ascending aortic aneurysm which contained a dissection flap. There were very poor left ventricular function and grossly elevated filling pressures. Bypass was established via bicaval venous cannulation and the underside of the aortic arch. The aorta was cross clamped just proximal to the innominate artery, distal to the dissection flap and at a point where the aorta measured approximately 4 cm in diameter. Straightforward removal of the recipient's heart was performed whilst he was cooled to 18 C. In the meanwhile the left lung could be reflected into the now empty pericardial cavity and the area of the coarctation exposed. A good view was obtained although it was not feasible to place a clamp proximally as the arch of the aorta disappeared out of the field of vision.

Once the patient was stable at 18 C circulatory arrest was instituted. The coarctation was incised vertically. It had been planned to place an on laid patch but this was clearly not going to be possible so the coarctation area was excised. A short length of 24 mm Vascutek graft was then sutured in an end to end fashion between the two cut ends of the aorta. Total circulatory arrest time for this rather awkward anastomosis was 36 minutes. After initial deairing the circulation was restored and the patient rewarmed. A relatively straightforward orthotopic cardiac transplant was performed with bicaval cannulation. The aortic anastomosis was on to the donor aorta, obliquely divided at the level of the innominate artery. Donor heart ischaemic time was 78 minutes. Post-operatively he had periods of profound vasodilatation and subsequent confusion. He was ventilated for 8 days and underwent a tracheostomy on day five. He eventually made a full recovery with no residual neurological deficit and was discharged home at 28 days. There was no measurable difference, by non invasive techniques, between upper and lower limb blood pressure at that time.

## Discussion

10% of the patients with thoracic coarctation present after the age of 40 [1]. In these adults there is often associated medical pathology, with 40% of those presenting over the age of 30 with associated cardiac conditions [1,2]. The link between coarctation of the aorta and the development of proximal dissection was first described by Abbott [3]. The aortic rupture, normally in the ascending aorta, is the cause of death in 20% of undiagnosed coarctation patients in a historical review [4].

The standard approach to this problem has either been staged procedures (repair of a coarctation followed by repair of cardiac anomaly) or a non anatomical bypass from the ascending aorta to descending or supra-coeliac abnormal aorta [5,6]. The commonest simultaneous procedure is an aortic valve replacement. In one patient the combination of extra anatomical repair and heart transplant was described [7]. The third option would be balloon dilatation of the coarctation with subsequent repair of the cardiac anomaly. This was considered in our case but decided against because of the close relationship to an aneurysmal intercostal artery.

A trans-sternal anatomic repair of the coarctation and associated cardiac defects has been widely reported in children [8] but rarely in adults. The left lung severely impairs access to the area of the coarctation. In this instance a combination of hyporthermic circulatory arrest to exclude the need to clamp the aorta excluding the need to expose and then clamp the aorta, coupled with the ability to reflect the left lung medially into the empty pericardial space (following recipient cardiectomy), made the combined procedure that of choice. The combination of circulatory arrest and post-operative hypertension probably delayed a full neurological recovery but the eventual outcome was excellent.

## Competing interests

The authors declare that they have no competing interests.

## Authors' contributions

HP carried out the literature research, participated in the sequence alignment and drafted the manuscript, BA helped with the construction of the manuscript, AS was helpful in literature review and the "discussion" part, of the paper and JD assist in the development of the manuscript and advised on valuable amendments. The authors read and approved the final manuscript.

## Consent

Written informed consent was obtained from the patient for publication of this case report and accompanying images. A copy of the written consent is available for review by the Editor-in-Chief of this journal.
